# Social and health factors associated with unfavourable treatment outcomes in children and adolescents with drug-sensitive tuberculosis in Brazil: a national retrospective cohort study

**DOI:** 10.1016/j.lana.2024.100938

**Published:** 2024-11-13

**Authors:** Victor Santana Santos, Jamile Rodrigues Cosme de Holanda, Ruy Dantas Silveira Gois-Neto, Ethel Leonor Noia Maciel, Fernanda Dockhorn Costa Johansen, José Nildo de Barros Silva-Júnior, Wesley Adson Costa Coelho Correio, José Roberto Lapa e Silva, José Rodrigo Santos Silva, Ricardo Queiroz Gurgel, Tom Wingfield

**Affiliations:** a Health Sciences Graduate Program, Federal University of Sergipe, Aracaju, Brazil; b Department of Medicine, Federal University of Sergipe, Lagarto, Brazil; c Department of Nursing, Federal University of Espírito Santos, Vitória, Brazil; d Secretaria de Vigilância em Saúde e Ambiente, Ministério da Saúde, Brasília, DF, Brazil; e FACENE, Mossoró, Brazil; f Graduate Program of Clinical Medicine, Federal University of Rio de Janeiro and Graduate Program of Medical Sciences, Fluminense Federal University, Brazil; g Department of Statistics and Actuarial Sciences, Federal University of Sergipe, São Cristóvão, Brazil; h Department of Medicine, Federal University of Sergipe, Aracaju, Brazil; i Centre for Tuberculosis Research, Departments of International Public Health and Clinical Sciences, Liverpool School of Tropical Medicine, Liverpool, UK; j Department of Global Public Health, Karolinska Institutet, Stockholm, Sweden; k Tropical and Infectious Disease Unit, Liverpool University Hospitals NHS Foundation Trust, Liverpool, UK

**Keywords:** Tuberculosis, Social and health factors, Unfavourable treatment outcomes, Child and adolescent health

## Abstract

**Background:**

Although tuberculosis (TB) poses a significant global health threat to children and adolescents, there is limited information on the factors associated with TB treatment outcomes in this group. This study investigated the social and health factors associated with unfavourable treatment outcomes in children and adolescents with TB in Brazil, a high TB burden country.

**Methods:**

We conducted a population-based national retrospective cohort study of children (0–9 years) and adolescents (10–17 years) with TB in Brazil notified to the national *Sistema de Informação de Agravos de Notificação* (Sinan) from Jan 1, 2001, to Dec 31, 2022. Unfavourable treatment outcomes were defined as loss to follow-up, treatment failure, and death. Logistic regression and multinomial models examined the association between social and health factors, unfavourable treatment outcomes overall, and loss to follow-up and death, respectively.

**Findings:**

A total of 88,270 children and adolescents with TB were included of whom 25,600 (30.6%) had healthcare worker-supervised directly observed therapy (DOT). Of these, 9303 (10.5%) individuals experienced unfavourable TB treatment outcomes. For children, HIV infection (adjusted Odds Ratio 2.4, 95% confidence interval 1.9–3.1) and did not receive DOT (2.3, 1.9–2.7) were associated with unfavourable treatment outcomes. For adolescents, alcohol use (1.6, 1.2–2.0), illicit drug use (4.2, 3.4–5.1), tobacco use (1.6, 1.3–2.1), HIV infection (2.7, 2.2–3.4), and not receiving DOT (2.6, 2.3–2.9) were associated with unfavourable TB treatment outcome. Receiving social protection through government cash transfers protected against death (0.5, 0.3–0.9).

**Interpretation:**

In Brazil, TB treatment success rates were comparable to WHO End TB Strategy targets (90%). Substance use, HIV infection, and the absence of supervised treatment were the main factors associated with unfavourable treatment outcomes. Strategies to improve equity of TB treatment outcomes in this vulnerable group, including integrated HIV-TB services, DOT in healthcare facilities or communities, and holistic, person-centred healthcare and social protection, should be evaluated.

**Funding:**

10.13039/501100000276Department of Health and Social Care (DHSC), the 10.13039/501100020171Foreign, Commonwealth & Development Office (FCDO), the 10.13039/501100000265Medical Research Council (MRC) and 10.13039/100004440Wellcome, UK.

**Research in context**

**Evidence before this study**

We searched PubMed, Scopus, Web of Science, Embase, and Google Scholar on 20 September 2023 for published studies that evaluated children and adolescents with TB in Brazil without language restriction. We used the search terms “Tuberculosis”, “Children”, “Adolescents”, “Unfavourable Treatment Outcome”, “Adverse Treatment Outcome”, “Loss to Follow Up”, “Death”, “Brazil”, and related synonyms. Few studies on children and adolescents were identified, most of which were performed at the state level and the single national study was focused on young adults (10–24 years old). There were no national-level studies identified that evaluated the social and health factors associated with unfavourable TB treatment outcomes among children (0–9 years old) in Brazil.

**Added value of this study**

To our knowledge, this study represents the first nationally representative analysis of the characteristics of children and adolescents (aged 0–17 years) with TB in Brazil and an assessment of the factors associated with unfavourable TB treatment outcomes in this age group. The study showed a rate of TB treatment success of 89.5% in this age group, similar to the WHO target of 90%. It was notable that less than one-third (31%) of children and adolescents with TB had healthcare worker-supervised Directly Observed Therapy (DOT). Non-white children and adolescents, with HIV infection, with the pulmonary clinical form and without treatment supervision and adolescents who used alcohol, illicit drug, or tobacco were more likely to have an unfavourable treatment outcome.

**Implications of all the available evidence**

The implications of all the evidence we have gathered underscore that TB care and prevention policy and practice in Brazil could benefit from the evaluation of integrated TB-HIV services and strengthening the partnership between the Family Health Strategy and the School Health Programme. Such a comprehensive approach would mitigate the impact of these intersecting factors on TB outcomes. Additionally, it emphasizes the importance of sustained support and adherence during treatment, highlighting the need to advocate for the incorporation of person-centred Directly Observed Therapy (DOT) or Community-Based DOT (CB-DOT). These holistic dimensions that go beyond pills and tests to address the broader social and behavioural determinants of TB and adverse TB treatment outcomes in Brazil is likely to enhance the care and prevention of this devastating disease of poverty.

## Introduction

Tuberculosis (TB) remains a major public health problem worldwide, with an estimated 1.1–1.7 million deaths and more than 10 million incident cases per year.^1^^,^^2^ The World Health Organization (WHO) set a target of ≥90% TB treatment success rate to achieve their goal of a 75% reduction in deaths from TB by 2025, compared to 2015.^1^^,^^2^ However, the COVID-19 pandemic negatively impacted TB control programmes, affecting TB prevention, case detection, and management, particularly in low- and-middle-income countries.^1^^,^^3^

The impact of TB on children and adolescents is a particular challenge. Paediatric TB can be difficult to diagnose, especially in the context of HIV infection.^4^^,^^5^ Approximately 10% of people with TB are under 15 years old.^1^^,^^2^ The rate of TB in this age group varies across regions, with higher prevalence in areas with higher background TB prevalence among adults.^1^ Young children most commonly become infected with TB following exposure to an adult with TB in their household.^5^ However, in endemic areas, significant transmission occurs outside of the household.^6^ Adolescents have the potential to contribute to TB transmission due to a wide and active social network. Adolescents are more likely to have pulmonary than extrapulmonary TB, with proportions similar to adults with TB, and account for a considerable proportion of new TB notifications wordwide.^7^^,^^8^ In addition, there is a concerning trend of high loss-to-follow-up rates during TB treatment among adolescents compared to other age groups, which not only impacts on morbidity and mortality rates but also increases the risk of development of drug-resistant TB (DR-TB).^9^ Furthermore, TB amongst adolescents often indicates recent and ongoing transmission within the community.^1^^,^^4^^,^^5^

The WHO's 2013 and 2018 roadmap towards ending TB has ignited optimism for expediting initiatives aimed at eradicating TB in children and adolescents by guaranteeing their utmost priority in all TB care and prevention approaches.^7^ However, childhood and adolescence are often overlooked in national TB strategies, with TB treatment programmes mostly focusing on adults and prevention strategies prioritising children under 5 years.^5^^,^^7^^,^^10^ Therefore, the WHO's 2023 roadmap to end TB among children and adolescents retains a continued emphasis on paediatric TB, alongside highlighting the significance of addressing TB in adolescents, who exhibit high TB rates, and possess unique age-related needs that warrant attention for enhanced outcomes.^7^

Brazil, which is among the 30 countries with the highest TB burden,^1^ had a 21% drop in notifications in the 5 to 14 age group and 28% in children under 5 years between 2019 and 2020 as a result of the COVID-19 pandemic.^11^ The Ministry of Health of Brazil also reported that there was a national reduction in treatment success rates from 70% to 67% amongst the general population, and from 74% to 71% among children and adolescents, between 2019 and 2020.^11^ In response to these challenges, Brazil has endeavoured to mitigate the damage caused in recent years by adopting and promoting actions to expand access to early diagnosis and increase rates of treatment success. It is, therefore, necessary to characterise the factors associated with the unfavourable outcome of TB treatment among children and adolescents to better guide public health actions.

A previous national cohort study focused on young individuals (10–24 years old) with TB in Brazil from 2015 to 2018 and showed observed treatment success rates below the targets set by the World Health Organization's End TB Strategy.^12^ Nearly one-fifth of the young people in this study experienced unfavourable outcomes, attributed predominantly to homelessness, HIV, and illicit drug use. Conversely, young people who received government cash transfers were less likely to have unfavourable outcomes, highlighting a potential protective impact.^12^ However, no nationally representative longitudinal studies have been conducted assessing the factors linked to unfavourable treatment outcomes among children and adolescents with TB in Brazil. Conducting such research is crucial, not only from a human rights standpoint but also because unfavourable TB treatment outcomes in children and adolescents heighten the risk of subsequent TB transmission, TB-related morbidity, mortality, and adverse socioeconomic consequences. Additionally, studies such as these can assist decision-makers and TB control programmes in designing and implementing better-targeted public policies.

Therefore, in this nationwide population-based cohort, we investigated the social and health factors associated with unfavourable treatment outcomes among children and adolescents with new TB diagnoses in Brazil.

## Methods

### Study design and participants

We conducted a population-based retrospective cohort study of children and adolescents with TB in Brazil notified to the System of Information for Notification of Diseases (Sinan, *Sistema de Informação de Agravos de Notificação*). Sinan is a de-identified national public domain database established by the Brazilian Ministry of Health for the surveillance of notifiable diseases, including TB. TB notifications are compulsory, and Sinan receives notifications from all public and private health centres in Brazil. The Sinan database undergoes rigorous validation processes to ensure data integrity and completeness, contributing to its robustness as a reliable source for infectious disease research. Notification data includes sociodemographic and clinical features.

Our analysis included all individuals aged 0–17 years, referred to as children (0–9 years) and adolescents (10–17 years), notified on Sinan with a new diagnosis of TB from Jan 1, 2001, to Dec 31, 2022. Data were obtained in September 2023. We used the definition of children and adolescents adopted by the Brazilian authorities,^13^ and in related studies.^14^^,^^15^ We excluded individuals who were notified on Sinan due to TB recurrence or return following loss to follow–up, those who had a TB diagnosis at post-mortem investigation, and those who had a missing notification status. We also excluded individuals who had missing treatment outcomes (still undergoing treatment), those whose outcome was recorded as transfer to other healthcare centres, or those whose diagnosis changed during TB treatment. Since the outcome of individuals with drug-resistant TB is not recorded in the Sinan but in a separate registry (Special Treatment of TB Information System), we excluded individuals who had drug-resistant TB.

### Study area and context

Brazil has a geographic area of 8.5 million square kilometres, and ∼210 million population, of whom approximately 60 million are less than 20 years old. The illiteracy rate in people ≥15 years old is 6.6%, the Human Development Index (HDI) is 0.765 and infant mortality is 12.4 deaths per 1000 live births. There is marked socioeconomic and health infrastructure variation across the North, Northeast, Central-West, Southeast and South Brazilian regions. The North and Northeast regions often exhibit higher rates of poverty and lower HDI scores compared to the more economically developed Southeast and South regions. This economic discrepancy is reflected in health infrastructure, where the latter regions tend to have more advanced healthcare facilities, higher physician densities, and better-equipped hospitals. The Central-West region serves as a transition zone, featuring both economic areas and regions with more limited resources. Socio-economic and health indicators in this region vary widely, and the availability of healthcare services may depend on the specific state or locality. The Southeast region, encompassing major urban centres, boasts a more developed health infrastructure and higher HDI. Access to specialised medical services, well-equipped hospitals, and a higher concentration of healthcare professionals contribute to better health outcomes in this region. In the South, there is a similar pattern of higher socio-economic development and better health infrastructure. The region is known for its well-established healthcare facilities and services.

### Covariates and definitions

We extracted specific social and health indicators from Sinan that delineate three distinct facets of vulnerability—namely, individual, social, and institutional vulnerability.^16^ These indicators encompassed.

A) Individual vulnerability variables: sex (male/female), age group, ethnicity, tobacco consumption, alcohol use, illicit drug use, and HIV or AIDS status. For children and adolescents, any exposure or use of alcohol, illicit drugs and tobacco was considered misuse. We consider information for these variables as provided by Sinan. Race and ethnicity were self–identified and based on the five classifications defined by the Brazilian Institute of Geography and Statistics: White (*Branco*), Black (*Preto*), Brown (*Pardo*), Asian (*Amarelo*), or Indigenous (*Indígena*).
B) Social vulnerability variables: geographical regions, participation in social protection programmes, homelessness, and deprivation of liberty. Brazil is divided into five geopolitical regions (North, Northeast, Central–West, Southeast, and South) which have different social and economic indicators, health system capacity, and coverage. Deprivation of liberty refers to the situation in which the child or adolescent has been institutionalised in shelters or institutions under the guardianship of the state.
C) Institutional vulnerability variables: chest radiographic evidence of TB, initial smear microscopy results, clinical form, and directly observed therapy (DOT). In Brazil, for operational purposes, DOT is achieved when the medication is taken at least three times per week throughout the treatment, under the supervision of a healthcare professional. At the end of the treatment, the individual must have completed a minimum of 24 doses during the intensive phase and 48 doses during the maintenance phase.

### Outcomes

The primary outcome of the study was the treatment outcome of TB, which was represented as a binary variable, categorised as either “favourable” or “unfavourable” according to current Brazilian Ministry of Health guidelines.^17^ Favourable treatment outcomes were defined as outcomes of “treatment completion” and “cure”. For people with pulmonary TB, “cure” was characterised as a clinician-led discharge from TB care upon the completion of TB treatment and a minimum of two consecutive negative sputum smear tests prior to the end of treatment. In the case of people with extrapulmonary TB, “cure” was defined as either the successful completion of TB treatment or the presence of clinical improvement and radiological resolution.^17^ Unfavourable treatment outcomes were characterised by one of the following conditions: (1) “loss to follow–up,” signifying treatment disruption for 30 consecutive days or more; (2) “death” by TB during the TB treatment; (3) “treatment failure”, which was defined as the persistence of a positive sputum smear or culture for *M. tuberculosis* at or beyond the fifth month of therapy. The final recorded TB treatment outcomes were documented within nine months of the initial notification on Sinan, or 15 months for cases involving the central nervous system.^17^

In addition, we analysed the social and health factors associated with each of the components of unfavourable TB treatment outcome (loss to follow–up, treatment failure, and death) vs favourable TB treatment outcome.

### Data analysis

The data analysed comprised all children and adolescents aged 0–17 years with TB registered in the Sinan database from Jan 1, 2001, to Dec 31, 2022. Complete data were not available for all variables. We did not impute missing data for sex, ethnicity, Brazilian regions, and clinical features of TB. However, missing values were interpreted as the absence of government cash transfer, deprivation of liberty, homelessness, alcohol use, illicit drug use, tobacco use, HIV, diabetes, and treatment supervision provided. Categorical variables are reported as absolute and relative frequencies. Factors associated with the unfavourable outcome were established using the odds ratio (OR) with 95% confidence intervals (CI).

Multivariate logistic regression was used to identify factors independently associated with unfavourable outcomes. Similar to the study by Chenciner and colleagues (2021),^12^ in the adjusted models, all missing data a priori (complete-case analysis) were excluded. To assess the robustness of the findings in relation to the exclusion of missing data, we managed a post-hoc analysis using a missing data indicator, incorporating missing data as a category within the multiple logistic regression models (data shown in Appendix).^12^ Additionally, we conducted pre-defined stratified analyses to investigate potential variations in factors associated with unfavourable treatment outcomes between two age groups: children, defined as individuals aged 0–9 years, and adolescents, defined as those aged 10–17 years. Finally, multinomial logistic regression was used to evaluate the different factors of loss to follow-up and death, with successful treatment as the reference outcome category. Analyses of the outcome of “treatment failure” were suppressed due to the small number of events (n = 25). In all models, the calendar year was included as a control covariate. For all analyses, P values less than 0.05 were considered statistically significant. Data were analysed using R software version 4.3.1 (R Core Team 2023).

### Ethical considerations

As data in Sinan are deidentified and publicly available, institutional review board approval and informed consent were not required.

### Role of funding source

The study's funder had no role in the study design, data collection, management, analysis, interpretation, or writing of the report.

## Results

**Fig. 1:**
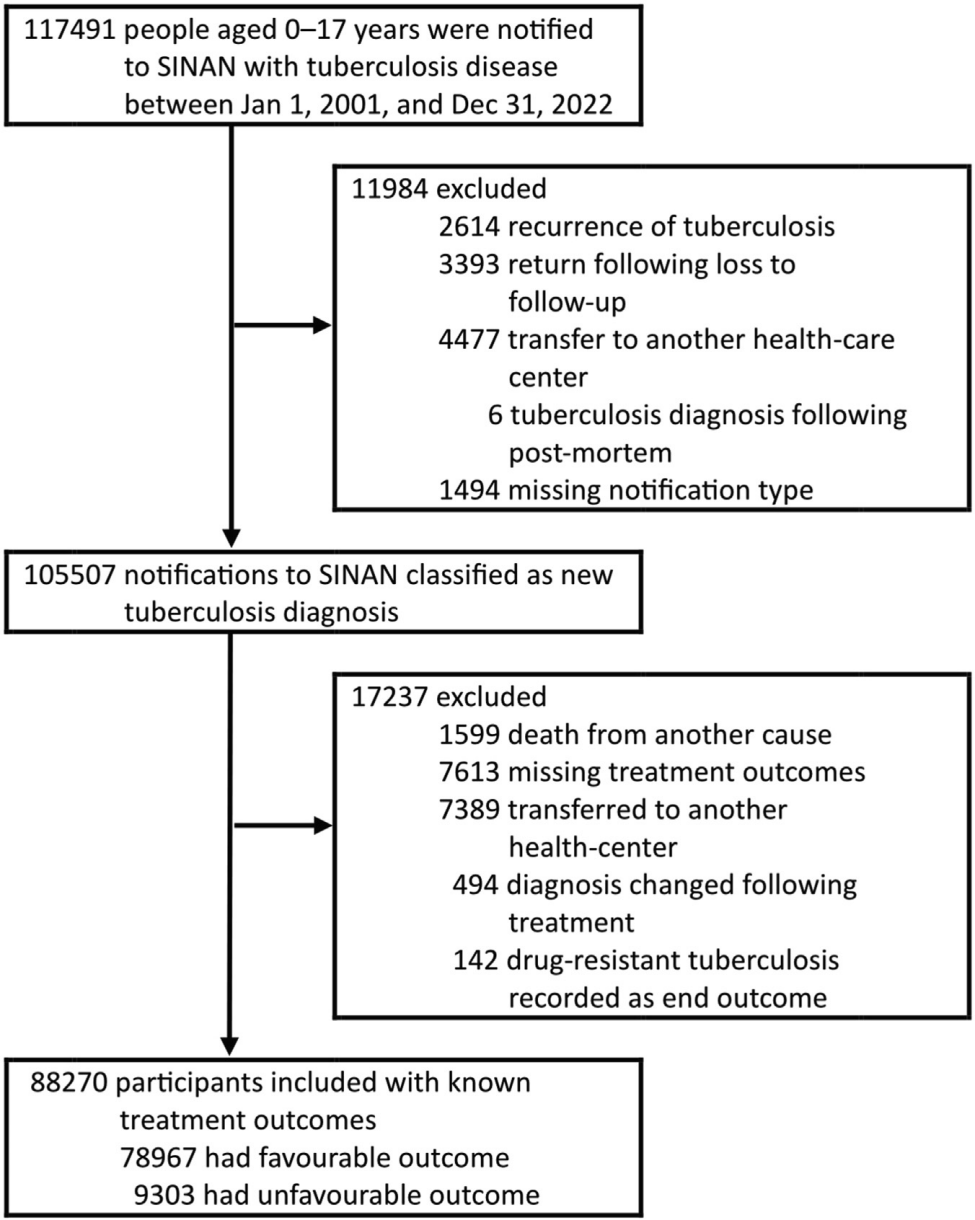
Flow chart for selecting study participants.

A total of 117,491 children and adolescents with TB were notified on Sinan between January 2001 and December 2022, of which 88,270 met the eligibility criteria and were then included in the analysis (Fig. 1).

Table 1 describes the sociodemographic and clinical characteristics of the individuals included. Predominantly, the cohort consisted of adolescents aged 10–17 years (n = 60,110; 68%), males (n = 45,904; 52%), and individuals self–identifying as having Brown (Pardo) race/ethnicity (n = 35,847; 50.2%). Most participants resided in the Southeast region (n = 38,800; 44%). Only 3143 (3.8%) individuals were directly enrolled in government cash transfer programs. A total of 415 (0.5%) children and adolescents were deprived of liberty, and 145 (0.2%) were documented as homeless. The prevalence of alcohol, illicit drug, and tobacco use was 2%, 1.5%, and 1.4%, respectively. eTable 1 in the appendix shows the age distribution for exposure to alcohol, illicit drugs and tobacco.

**Table 1:** Sociodemographic and clinical characteristics of the study participants (excluding missing data).

Variable	Children (n = 28,160) N (%)	Adolescents (n = 60,110) N (%)	All (n = 88,270) N (%)
**Sex (n** = **88,251)**			
Male	15,587 (55.4)	30,317 (50.4)	45,904 (52.0)
Female	12,561 (44.6)	29,786 (49.6)	42,347 (48.0)
**Race/Ethnicity (n** = **71,346)**			
Asia	175 (0.8)	526 (1.1)	701 (1.0)
Indigenous	1520 (6.8)	1318 (2.7)	2838 (4.0)
Brown (*Pardo*)	10,638 (47.8)	25,209 (51.3)	35,847 (50.2)
Black	2384 (10.7)	6225 (12.7)	8609 (12.1)
White	7525 (33.8)	15,826 (32.2)	23,351 (32.7)
**Region (n** = **88,195)**			
Central West	1806 (6.4)	2145 (3.6)	3951 (4.5)
Northeast	7464 (26.5)	16,511 (27.5)	23,975 (27.2)
North	3677 (13.1)	8832 (14.7)	12,509 (14.2)
South	2773 (9.9)	6187 (10.3)	8960 (10.2)
Southeast	12,402 (44.1)	26,398 (43.9)	38,800 (44.0)
**Government cash transfer (n** = **83,666)**			
Yes	1041 (3.9)	2102 (3.7)	3143 (3.8)
No	25,669 (96.1)	54,854 (96.3)	80,523 (96.2)
**Deprivation of liberty (n** = **86,801)**			
Yes	199 (0.7)	216 (0.4)	415 (0.5)
No	27,590 (99.3)	58,796 (99.6)	86,386 (99.5)
**Homelessness (n** = **86,747)**			
Yes	60 (0.2)	85 (0.1)	145 (0.2)
No	27,691 (99.8)	58,911 (99.9)	86,602 (99.8)
**Alcohol use (n** = **69,126)**			
Yes	468 (2.1)	893 (1.9)	1361 (2.0)
No	21,497 (97.9)	46,268 (98.1)	67,765 (98.0)
**Illicit drug use (n** = **86,507)**			
Yes	156 (0.6)	1122 (1.9)	1278 (1.5)
No	27,543 (99.4)	57,686 (98.1)	85,229 (98.5)
**Tobacco use (n** = **86,604)**			
Yes	288 (1.0)	907 (1.5)	1195 (1.4)
No	27,435 (99.0)	57,974 (98.6)	85,409 (98.6)
**HIV (n** = **45,171)**			
Yes	1017 (8.3)	1055 (3.2)	2072 (4.6)
No	11,212 (91.7)	31,887 (96.8)	43,099 (95.4)
**Diabetes (n** = **68,791)**			
Yes	274 (1.3)	410 (0.9)	684 (1.0)
No	21,570 (98.7)	46,537 (99.1)	68,107 (99.0)
**Clinical feature of tuberculosis (n** = **88,269)**			
Pulmonary	20,576 (73.1)	48,935 (81.4)	69,511 (78.8)
Pulmonary + Extrapulmonary	1127 (4.0)	1447 (2.4)	2574 (2.9)
Extrapulmonary	6457 (22.9)	9727 (16.2)	16,184 (18.3)
**Chest radiography findings (n** = **74,110)**			
Abnormal	20,452 (86.1)	46,685 (92.7)	67,137 (90.6)
Normal	3308 (13.9)	3665 (7.3)	6973 (9.4)
**Baseline smear microscopy (n** = **86,089)**			
Positive	3766 (13.9)	29,885 (50.6)	33,651 (39.1)
Negative	4186 (15.5)	12,515 (21.2)	16,701 (19.4)
No result available	19,120 (70.6)	16,617 (28.2)	35,737 (41.5)
**DOT treatment supervision provided (n** = **83,664)**			
Yes	7527 (28.3)	18,073 (31.7)	25,600 (30.6)
No	19,035 (71.7)	39,029 (68.4)	58,064 (69.4)
**Outcome (n** = **88,270)**			
Loss to follow up	2529 (9.0)	5851 (9.7)	8380 (9.5)
Treatment failure	5 (0.1)	20 (0.1)	25 (0.1)
Death	470 (1.7)	428 (0.7)	898 (1.0)
Cure or treatment completion	25,156 (89.3)	53,811 (89.5)	78,967 (89.5)

A total of 2072 (4.6%) individuals were living with HIV. Most individuals within this study were diagnosed with pulmonary TB (n = 69,511; 78.8%) and exhibited abnormal chest radiography findings (n = 67,137; 90.6%) (Table 1). The reporting of smear microscopy results was hampered by missing data; nonetheless, among those with available data, the majority had a positive baseline smear microscopy result (n = 33,651). A total of 25,600 (30.6%) individuals had DOT. There was no difference in missing data between favourable and non-favourable outcomes (eTable 2 in Appendix).

A total of 9303 (10.5%) individuals with TB experienced unfavourable treatment outcomes, of whom 8380 (90.1%) were lost to follow–up, 25 (0.2%) had treatment failure, and 898 (9.7%) died during treatment.

Univariate analyses revealed that specific participant groups exhibited an increased likelihood of unfavourable treatment outcomes: male individuals (OR 1.2, 95% CI 1.1–1.2), Asian (1.4, 1.1–1.8) or Brown (1.5, 1.4–1.6) or Black (1.7, 1.6–1.8) compared to self–identifying as White race/ethnicity, those living in the Northeast (1.2, 1.1–1.3) or North (1.2, 1.1–1.3) or Southeast (1.1, 1.0–1.2) regions compared to the South region. Additional factors associated with unfavourable treatment outcome included currently deprived of liberty (1.7, 1.3–2.2), homelessness (3.7, 2.6–5.3), alcohol use (3.0, 2.6–3.4), illicit drug use (5.1, 4.5–5.7), tobacco use (3.8, 3.3–4.3), HIV infection (3.0, 2.6–3.3), and lack of treatment supervision (2.1, 2.0–2.2) (Table 2).

**Table 2:** Univariate analysis of social and health factors associated with unfavourable tuberculosis treatment among children and adolescents.

Variable	Unfavourable treatment outcome N (%)	Favourable treatment outcome N (%)	Crude OR (95% CI)	P–value
**Age group**				
Children	3004 (32.3)	25,156 (31.9)	Reference	
Adolescents	6299 (67.7)	53,811 (68.1)	1.0 (0.9–1.0)	0.402
**Sex**				
Male	5164 (55.5)	40,740 (51.6)	1.2 (1.1–1.2)	0.000
Female	4136 (44.5)	38,211 (48.4)	Reference	
**Race/Ethnicity**				
Asia	77 (1.0)	624 (1.0)	1.4 (1.1–1.8)	0.005
Indigenous	247 (3.3)	2591 (4.1)	1.1 (1.0–1.3)	0.190
Brown (*Pardo*)	4112 (55.5)	31,735 (49.6)	1.5 (1.4–1.6)	0.000
Black	1110 (15.0)	7499 (11.7)	1.7 (1.6–1.8)	0.000
White	1862 (25.1)	21,489 (33.6)	Reference	
**Region**				
Central West	362 (3.9)	3589 (4.5)	1.0 (0.8–1.1)	0.596
Northeast	2658 (28.6)	21,317 (27.0)	1.2 (1.1–1.3)	0.000
North	1394 (15.0)	11,115 (14.1)	1.2 (1.1–1.3)	0.000
Southeast	4030 (43.4)	34,770 (44.1)	1.1 (1.0–1.2)	0.011
South	849 (9.1)	8111 (10.3)	Reference	
**Government cash transfer**				
Yes	331 (3.8)	2812 (3.7)	1.0 (0.9–1.1)	0.740
No	8319 (96.2)	72,204 (96.3)	Reference	
**Deprivation of liberty**				
Yes	69 (0.8)	346 (0.4)	1.7 (1.3–2.2)	0.000
No	9057 (99.2)	77,329 (99.6)	Reference	
**Homelessness**				
Yes	44 (0.5)	101 (0.1)	3.7 (2.6–5.3)	0.000
No	9059 (99.5)	77,543 (99.9)	Reference	
**Alcohol use**				
Yes	345 (4.7)	1016 (1.6)	3.0 (2.6–3.4)	0.000
No	6964 (95.3)	60,801 (98.4)	Reference	
**Illicit drug use**				
Yes	462 (5.1)	816 (1.1)	5.1 (4.5–5.7)	0.000
No	8590 (94.9)	76,639 (98.9)	Reference	
**Tobacco use**				
Yes	359 (4.0)	836 (1.1)	3.8 (3.3–4.3)	0.000
No	8696 (96.0)	76,713 (98.9)	Reference	
**HIV infection**				
Yes	450 (10.8)	1622 (4.0)	3.0 (2.6–3.3)	0.000
No	3703 (89.2)	39,396 (96.0)	Reference	
**Diabetes**				
Yes	79 (1.1)	605 (1.0)	1.1 (0.9–1.4)	0.434
No	7186 (98.9)	60,921 (99.0)	Reference	
**Clinical feature of tuberculosis**				
Pulmonary	7381 (79.3)	62,130 (78.7)	1.1 (1.1–1.2)	0.000
Pulmonary + Extrapulmonary	400 (4.3)	2174 (2.8)	1.8 (1.6–2.0)	0.000
Extrapulmonary	1521 (16.4)	14,663 (18.6)	Reference	
**DOT treatment supervision provided**				
Yes	1605 (18.5)	23,995 (32.0)	Reference	
No	7065 (81.5)	50,999 (68.0)	2.1 (2.0–2.2)	0.000

Brazil, 2001 to 2022.

The multivariate regression complete case analysis is shown in Table 3. Children and adolescents self–declared as Asian (adjusted OR: 1.7, 95% CI: 1.1–2.6), Indigenous (1.6, 1.0–2.0), Brown (1.6, 1.5–1.8) and Black (1.9, 1.6–2.2) were more likely to have an unfavourable outcome than individuals self–declared as White. Children and adolescents living in the Southeast region (0.8, 0.7–0.9) were less likely to have an unfavourable treatment outcome compared to those living in the South region. Individuals who were exposed to alcohol (1.6, 1.3–2.0), illicit drugs (3.8, 3.1–4.6) and tobacco (1.6, 1.3–2.0) were also more likely to have an unfavourable treatment outcome. Other characteristics independently associated with unfavourable treatment outcomes were HIV infection (2.6, 2.2–3.0), TB presentation of pulmonary TB (1.2, 1.1–1.3) and TB pulmonary and extrapulmonary together (1.7, 1.3–2.1). Children and adolescents not having received DOT by healthcare workers had a 2.5-fold (2.3–2.7) higher odds of an unfavourable treatment outcome. Multivariate logistic regression using the missing indicators are showed in Appendix eTable 2.

**Table 3:** Multivariate logistic regression analysis of social and health factors associated with unfavourable tuberculosis treatment among children and adolescents.

Variable	Adjusted OR (95% CI)	P–value
**Age group**		
Children	Reference	
Adolescents	1.0 (0.9–1.1)	0.955
**Sex**		
Male	1.0 (0.9–1.1)	0.932
Female	Reference	
**Race/Ethnicity**		
Asia	1.7 (1.0–2.6)	0.028
Indigenous	1.6 (1.0–2.2)	0.001
Brown (*Pardo*)	1.6 (1.5–1.8)	0.000
Black	1.9 (1.6–2.1)	0.000
White	Reference	
**Region**		
Central West	0.8 (0.6–1.1)	0.142
Northeast	0.9 (0.8–1.1)	0.209
North	0.9 (0.8–1.0)	0.136
Southeast	0.8 (0.7–0.9)	0.000
South	Reference	
**Government cash transfer**		
Yes	0.9 (0.7–1.0)	0.059
No	Reference	
**Deprivation of liberty**		
Yes	1.0 (0.6–1.5)	0.930
No	Reference	
**Homelessness**		
Yes	1.6 (0.9–2.7)	0.108
No	Reference	
**Alcohol use**		
Yes	1.6 (1.3–2.0)	0.000
No	Reference	
**Illicit drug use**		
Yes	3.8 (3.1–4.6)	0.000
No	Reference	
**Tobacco use**		
Yes	1.6 (1.3–2.0)	0.000
No	Reference	
**HIV infection**		
Yes	2.6 (2.2–3.0)	0.000
No	Reference	
**Diabetes**		
Yes	1.2 (0.8–1.6)	0.461
No	Reference	
**Clinical feature of tuberculosis**		
Pulmonary	1.2 (1.1–1.3)	0.004
Pulmonary + Extrapulmonary	1.7 (1.3–2.1)	0.000
Extrapulmonary	Reference	
**DOT treatment supervision provided**		
Yes	Reference	
No	2.5 (2.3–2.7)	0.000

Brazil, 2001 to 2022.

Table 4 shows the results of the multivariate logistic regression according to age group (children and adolescents). We uncovered a multifaceted landscape of influence across different age groups that appeared distinct between children and adolescents. Children self-identifying with Asian (adjusted OR: 2.4, 95% CI: 1.1–5.2), Brown (*Pardo*) (1.5, 1.2–1.8) and Black (1.7, 1.3–2.2) race/ethnicity exhibited higher odds of unfavourable treatment outcomes compared to White children. Alcohol exposure (1.9, 1.2–2.8) and HIV infection (2.4, 1.9–3.1) were significantly associated with unfavourable treatment outcomes. Among clinical forms, those presenting with concurrent pulmonary and extrapulmonary TB had a higher odds ratio of unfavourable outcomes (1.9, 1.3–2.6) compared to those presenting with either pulmonary or extrapulmonary TB alone. Children not receiving DOT had 2.3-fold (1.9–2.7) higher odds of an unfavourable outcome.

**Table 4:** Multivariate logistic regression analysis of social and health factors associated with unfavourable tuberculosis treatment according to age group (children and adolescents).

Variable	Children		Adolescents	
	Adjusted OR (95% CI)	P–value	Adjusted OR (95% CI)	P–value
**Sex**				
Male	1 (0.9–1.2)	0.954	1 (0.9–1.1)	0.936
Female	Reference		Reference	
**Race/Ethnicity**				
Asia	2.4 (0.9–5.2)	0.042	1.5 (0.9–2.6)	0.127
Indigenous	1.4 (0.9–2.1)	0.138	1.7 (1.2–2.3)	0.003
Brown (*Pardo*)	1.5 (1.2–1.8)	0.000	1.7 (1.5–1.9)	0.000
Black	1.7 (1.3–2.2)	0.000	2 (1.7–2.3)	0.000
White	Reference		Reference	
**Region**				
Central West	0.7 (0.4–1)	0.068	0.9 (0.7–1.3)	0.725
Northeast	1.1 (0.8–1.5)	0.465	0.8 (0.7–1)	0.059
North	0.9 (0.6–1.2)	0.464	0.9 (0.7–1.1)	0.208
Southeast	0.7 (0.5–0.9)	0.001	0.8 (0.7–1)	0.009
South	Reference		Reference	
**Government cash transfer**				
Yes	0.9 (0.6–1.2)	0.383	0.8 (0.7–1)	0.081
No	Reference		Reference	
**Deprivation of liberty**				
Yes	0.5 (0.2–1.2)	0.147	1.4 (0.8–2.3)	0.200
No	Reference		Reference	
**Homelessness**				
Yes	2.2 (0.8–5.2)	0.087	1.4 (0.7–2.8)	0.306
No	Reference		Reference	
**Alcohol use**				
Yes	1.9 (1.2–2.8)	0.002	1.6 (1.2–2)	0.001
No	Reference		Reference	
**Illicit drug use**				
Yes	1.8 (1–3.1)	0.056	4.2 (3.4–5.1)	0.000
No	Reference		Reference	
**Tobacco use**				
Yes	1.6 (1–2.5)	0.064	1.6 (1.3–2.1)	0.000
No	Reference		Reference	
**HIV infection**				
Yes	2.4 (1.9–3.1)	0.000	2.7 (2.2–3.4)	0.000
No	Reference		Reference	
**Diabetes**				
Yes	0.9 (0.4–1.6)	0.679	1.4 (0.8–2.1)	0.196
No	Reference		Reference	
**Clinical feature of tuberculosis**				
Pulmonary	1.1 (0.9–1.3)	0.366	1.3 (1.1–1.4)	0.001
Pulmonary + Extrapulmonary	1.9 (1.3–2.6)	0.000	1.5 (1.1–2.1)	0.003
Extrapulmonary	Reference		Reference	
**Treatment supervision provided**				
Yes	Reference		Reference	
No	2.3 (1.9–2.7)	0.000	2.6 (2.3–2.9)	0.000

Brazil, 2001 to 2022.

For adolescents, the trends and associations differed but remained equally pertinent. Adolescents self–declared as Black (adjusted 95% CI: 2.0, 1.7–2.3), Brown (1.7, 1.5–1.9) and Indigenous (1.7, 1.2–2.3) were more likely to have an unfavourable outcome than those self–declared as White. Substance use, including alcohol consumption (1.6, 1.2–2.0), Illicit drug use (4.2, 3.4–5.1) and tobacco use (1.6, 1.3–2.1) and HIV infection (2.7, 2.2–3.4) were significantly associated with unfavourable treatment outcomes. Notably, adolescents with pulmonary TB had an adjusted odds ratio of 1.3 (1.1–1.4), and the combination of pulmonary and extrapulmonary forms posed an odds ratio of 1.5 (1.2–2.1). Adolescents not receiving supervised treatment had 2.6-fold (2.3–2.9) higher odds of an unfavourable outcome (Table 4). Multivariate logistic regression models estimating the association between characteristics of children and adolescents in Brazil and unfavorable TB treatment outcomes, utilizing Missing Indicator Analysis, are presented in Appendix eTable 3.

The results of the multinomial regression analysis for loss to follow-up and death are shown in Table 5. Children were less likely to experience loss to follow-up (0.9, 0.8–1.0) and death (0.9, 0.8–1.0) during TB treatment compared to adolescents. Across both children and adolescents: males demonstrated a significantly reduced risk of death compared to females (0.8, 0.6–0.9), but there was no gender difference observed for loss to follow-up (1.0, 0.9–1.1); and individuals who self-identified as Asian (1.8, 1.1–2.9), Brown (1.7, 1.5–1.9) and Black (2.0, 1.7–2.3) displayed an increased risk of loss to follow-up; whereas Indigenous (3.6, 2.2–6.1) and Brown (1.7, 1.3–2.3) individuals exhibited an elevated likelihood of death when compared to Whites. Individuals residing in the Southeast region were less likely to experience loss to follow-up in TB treatment compared to those in the South region. Children and adolescents receiving government cash transfers were less likely to experience the outcome of death compared to those who did not receive any government assistance or benefits. Homelessness was associated with a higher chance of death from TB (4.6, 1.6–13.3), but was not associated with loss to follow-up.

**Table 5:** Multinomial logistic regression model of factors associated with the different components of unfavourable outcomes in tuberculosis treatment among Brazilian children and adolescents.

Variable	Loss to follow up		Death	
	Adjusted RRR^a^ (95% CI)	P–value	Adjusted RRR^a^ (95% CI)	P–value
**Age group**				
Children	0.9 (0.8–1.0)	0.007	0.9 (0.8–1.0)	0.000
Adolescents	Reference		Reference	
**Sex**				
Male	1.0 (0.9–1.1)	0.387	0.8 (0.6–0.9)	0.033
Female	Reference		Reference	
**Race/Ethnicity**				
Asian	1.8 (1.1–2.9)	0.015	0.7 (0.1–5.3)	0.752
Indigenous	1.2 (0.9–1.7)	0.162	3.6 (2.2–6.1)	0.000
Brown (*Pardo*)	1.7 (1.5–1.9)	0.000	1.7 (1.3–2.3)	0.001
Black	2.0 (1.7–2.3)	0.000	1.1 (0.7–1.8)	0.621
White	Reference		Reference	
**Region**				
Central West	0.9 (0.7–1.1)	0.272	0.8 (0.4–1.6)	0.579
Northeast	0.9 (0.8–1.1)	0.189	1.1 (0.7–1.7)	0.548
North	0.8 (0.7–1.0)	0.064	1.3 (0.8–2.0)	0.225
Southeast	0.8 (0.7–0.9)	0.001	0.8 (0.6–1.2)	0.249
South	Reference		Reference	
**Government cash transfer**				
Yes	1.0 (0.8–1.1)	0.569	0.5 (0.3–0.9)	0.013
No	Reference		Reference	
**Deprivation of liberty**				
Yes	1.1 (0.7–1.7)	0.797	0.4 (0.1–3.2)	0.404
No	Reference		Reference	
**Homelessness**				
Yes	1.4 (0.8–2.5)	0.300	4.6 (1.6–13.3)	0.005
No	Reference		Reference	
**Alcohol use**				
Yes	1.6 (1.3–2.0)	0.000	2.4 (1.4–4.3)	0.003
No	Reference		Reference	
**Illicit drug use**				
Yes	4.2 (3.4–5.1)	0.000	1.1 (0.5–2.6)	0.779
No	Reference		Reference	
**Tobacco use**				
Yes	1.6 (1.3–2.1)	0.000	1.5 (0.7–3.1)	0.261
No	Reference		Reference	
**HIV infection**				
Yes	2.3 (2.0–2.7)	0.000	3.4 (2.5–4.8)	0.000
No	Reference		Reference	
**Diabetes**				
Yes	1.0 (0.6–1.5)	0.823	2.8 (1.4–5.6)	0.004
No	Reference		Reference	
**Clinical feature of tuberculosis**				
Pulmonary	1.3 (1.1–1.4)	0.000	0.7 (0.5–1.0)	0.021
Pulmonary + Extrapulmonary	1.3 (1.0–1.6)	0.081	3.4 (2.3–5.0)	0.000
Extrapulmonary	Reference		Reference	
**DOT treatment supervision provided**				
Yes	Reference			
No	2.6 (2.3–2.8)	0.000	1.8 (1.4–2.4)	0.000

Successful outcome (cure and treatment completion) was the reference outcome.

a RRR: Relative Risk Ratio.

The impact of substance use on treatment outcomes was also evident across both children and adolescent age groups. Alcohol consumption was associated with both an increased risk of loss to follow-up (1.6, 1.3–2.0) and death (2.4, 1.3–4.3). Individuals reporting illicit drug use (4.2, 3.4–5.1) or tobacco consumption (1.6, 1.3–2.1) were more likely to have a loss to follow-up, while there was no association with death for both factors. HIV infection significantly elevated the likelihood of loss to follow-up (2.3, 2.0–2.7) and death (3.4, 2.5–4.8). Pulmonary TB was associated with an increased risk of loss to follow-up (1.3, 1.1–1.4), whereas pulmonary associated to extra-pulmonary TB was linked to a substantially elevated risk of death (3.4, 2.3–5.0). Not receiving supervised treatment of TB was associated with both loss to follow-up (2.6, 2.3–2.8) and death (1.8, 1.4–2.4).

## Discussion

To the best of our knowledge, this is the first nationally representative study of unfavourable treatment outcomes in children and adolescents with TB in Brazil. We report on 88,270 individuals from all health facilities in the country seen over 22 years from 2001 to 2022. Our study population predominantly included male individuals, adolescents aged 10–17 years, individuals self-identifying with Black or Brown race/ethnicity, and individuals living in the Southeast region of the country, most of whom had pulmonary TB. The most common unfavourable treatment outcome was loss to follow-up. There was a low frequency of children and adolescents receiving government cash benefits. Supervised treatment with DOT was provided for only 31% of the population included. In addition, our findings showed that non-white children and adolescents, individuals with HIV infection, with the pulmonary clinical form and without treatment supervision and adolescents experiencing alcohol, illicit drug, or tobacco use were more likely to have an unfavourable treatment outcome. Finally, our findings show some variation in the factors associated with either loss to follow-up or death specifically.

In this cohort, an estimated 11% of children and adolescents with TB experienced an unfavourable treatment outcome. Such findings align with the prevalent range reported in existing literature, typically falling between 5% and 15%,^15^^,^^18^, ^19^, ^20^ and similar to WHO End TB Strategy benchmarks.^21^^,^^22^ Nevertheless, the rate of unfavourable treatment outcomes observed among children and adolescents in this study remains comparatively lower than the rates observed in individuals reported with TB in Brazil over the past decade (21%, between 2010 and 2020).^11^^,^^12^ These results collectively underscore the difficulties associated with treating TB in paediatric populations and emphasize the importance of ongoing efforts to improve treatment strategies.

The results of our study concerning race/ethnicity paralleled prior research,^12^ indicating that individuals who self-identified as Black or Brown or Indigenous ethnicity faced increased odds of experiencing loss to follow-up and death due to TB in Brazil when compared to those who self-identified as White. Racial disparities in TB treatment outcomes among non-white children and adolescents can be attributed to a complex web of social determinants of health in Brazil. These determinants encompass socioeconomic status, education, neighbourhood conditions, and access to healthcare. Minority communities often face disproportionate challenges in these areas, leading to barriers to TB prevention, diagnosis, and management.^8^ Limited access to healthcare facilities, unequal distribution of resources, and inadequate health insurance coverage often result in delayed diagnosis and treatment initiation and contribute to unfavourable results among Black/Brown children and adolescents with TB.

This study found that children and adolescents living in the southeast of Brazil had a better chance of a favourable outcome. Studies suggest that better TB treatment indicators in Southeast Brazil are due to factors including satisfactory access to treatment, a range of services, better social conditions like income and schooling.^23^, ^24^, ^25^

The early use of substances by adolescents reveals a reality of social vulnerability in their families.^26^ Children and adolescents of parents who misuse alcohol or illicit drugs have a higher risk of consuming these substances.^26^^,^^27^ As in the adult^28^^,^^29^ and young adult^12^ populations, this study revealed that adolescents using psychoactive substances and tobacco were more likely to have an adverse TB treatment outcome. This dual challenge of substance usage and TB poses a significant obstacle to public health efforts, emphasizing the need for targeted interventions to address these issues simultaneously. Initiatives that address substance abuse among adolescents can potentially improve TB treatment outcomes and reduce the burden of the disease in Brazil.

There is limited empirical evidence demonstrating the global prevalence of co-infection between TB and HIV among children and adolescents population, where estimates of HIV positivity range from 10% to 60% in younger children with TB.^30^ In this investigation, 1.7% of children and 3.6% of adolescents were living with HIV, and individuals with HIV exhibited roughly 2.6 times higher odds of an unfavourable outcome in TB treatment. Our finding of an association between HIV co-infection and an elevated likelihood of unfavourable TB treatment outcomes aligns with findings from other studies.^12^^,^^19^^,^^31^ A systematic review of prediction models for pulmonary TB outcomes in adults also identified HIV as a common predictor of unfavourable treatment results.^19^ Globally, TB is responsible for a quarter of HIV-related fatalities, and the management and care of children and adolescents dealing with TB-HIV co-infection, particularly those in advanced stages of HIV, poses considerable challenges.^1^^,^^18^^,^^32^ Enhanced accessibility to well-integrated TB and HIV services is imperative for enhancing outcomes for individuals living with HIV and diagnosed with TB, especially for affected children and adolescents.^22^

In this study, children and adolescents facing homelessness were 4.6-fold more likely to experience death during TB treatment. A previous national study including all age groups showed that the homeless population exhibited 2.9-fold higher rate of loss to follow-up and 2.5 times higher mortality compared to the general population.^33^ The association between homelessness and an increased likelihood of loss to follow-up in TB treatment is multidimensional. Homeless individuals usually face precarious living conditions, high social vulnerability, extreme poverty, lack of shelter and malnutrition. The transient and unstable nature of homelessness often makes it challenging for individuals to access healthcare services and maintain a consistent treatment regimen.^34^ Limited financial resources, and insufficient social support networks contribute to difficulties in attending regular medical appointments.^34^, ^35^, ^36^

In this study, less than one-third of children and adolescents received supervised treatment with DOT, and the absence of supervised treatment was associated with a higher probability of unfavourable outcomes of TB treatment. This finding is in line with other studies from diverse settings.^12^ A systematic review and meta-analysis showed that people receiving TB treatment supervision are more likely to complete their treatment.^9^ DOT is pivotal in ensuring the success of TB treatment programs and preventing loss to follow-up. This supervision is crucial, especially for children and adolescents, for several reasons. First, it plays an important role in ensuring adherence to medication regimens, thereby preventing the development of drug-resistant strains of the disease.^9^ Furthermore, the supervision of TB treatment is essential for tracking patient progress and addressing any side effects or complications that may arise during treatment.^9^ TB medications can induce side effects, and some patients may experience adverse reactions necessitating immediate medical attention. In addition, individuals experiencing adverse effects during TB treatment are more likely to abandon their treatment.^37^ By closely monitoring people with TB throughout treatment, healthcare providers can identify and manage these issues promptly, ensuring adherence and treatment completion. Additionally, supervision fosters a support system for patients. The psychological and emotional support provided by healthcare workers during supervision can serve as a motivating factor, reducing the likelihood of patients dropping out of treatment.^9^

Despite the recognised benefits of DOT, its implementation in facilities dedicated to TB treatment has been a challenge. Primary healthcare settings often face limited resources, including staff, infrastructure, and funding, which can make it difficult to allocate the necessary personnel and resources for DOT implementation.^38^ In remote or underserved areas, reaching patients to provide direct observation can be logistically challenging. Travelling long distances to visit patients can strain resources and time.^37^^,^^39^ Some patients may be reluctant to undergo DOT due to concerns about privacy and fear of social stigma associated with TB.^39^ In addition, most vulnerable patients may face difficulty in accessing and completing DOT.^39^ Thus, unconditional social and financial support may be the key to successful adherence to TB treatment.

Technology and mobile applications can play a significant role in facilitating the implementation of supervision during TB treatment.^9^^,^^40^ Mobile apps can enable patients to record their medication adherence by simply checking in when taking their medication. This data can be remotely monitored by healthcare providers, allowing them to track patient compliance without the need for physical presence.^40^^,^^41^ Mobile apps can send medication reminders to patients, ensuring they take their medication at the correct times.^42^^,^^43^ Technology allows for efficient and accurate data collection, which is essential for monitoring patient progress and program effectiveness.^40^^,^^42^ Applications can collect real-time data on patient adherence, side effects, and treatment outcomes, making it easier for healthcare providers to tailor interventions and address emerging issues promptly.^40^^,^^42^ Finally, patients can use apps to report any side effects, concerns, or difficulties they encounter during treatment. In November 2023, the Brazilian Ministry of Health began recommending the use of digital technologies for DOT for people with TB.^44^

A low proportion of children and adolescents reported receiving cash transfers from the government. Nevertheless, children and adolescents receiving government cash transfers were less likely to experience death compared to those who did not receive any government assistance or benefits. Previous studies have shown the importance of the cash transfer programmes adopted by the Brazilian government (i.e., *Bolsa Família*) in improving TB treatment outcomes.^12^^,^^45^, ^46^, ^47^, ^48^ By providing direct cash transfers to low-income families, government cash transfer programmes help mitigate the economic burden that challenges treatment adherence and completion. This financial support ensures that individuals can afford transportation to healthcare facilities, cover essential costs like nutritious food, and maintain a stable living environment, all of which are essential for TB patients to adhere to their treatment regimen.^49^, ^50^, ^51^ However, *Bolsa Família* alone is not enough to mitigate the catastrophic costs of people with TB and their households and defray further impoverishment. Therefore, extra social protection must be adopted in a non-conditional way to assist people affected by TB and thus provide increased adherence to treatment, especially for underserved groups including children and adolescents.^49^

This cohort study assessed a large sample size of children and adolescents with TB, allowing a comprehensive analysis of the social and clinical factors associated with unfavourable treatment outcomes. Nevertheless, our findings should be interpreted with caution. The data were obtained from a surveillance information system and therefore only represent children and adolescents who accessed the health system and may underrepresent children and adolescents from areas with limited access to health services. Consequently, cases in poor communities could be missing and undetected, which would bias the detection rates from these areas. Individuals with TB who had missing data regarding their race/ethnicity, HIV status, or using alcohol, illicit drugs and tobacco exhibited a notably higher likelihood of experiencing unfavourable TB treatment outcomes. Consequently, the omission of these missing values may have led to an underestimation of the observed associations for these specific variables of interest. Similarly, missing data for variables such as government cash transfers may have underestimated their association with better treatment outcomes. We used complete-case analysis instead of an analytical imputation method, as it is simpler to apply and assumes that missing data occur completely at random. The Sinan has no socioeconomic status, limiting our ability to better understand the influence of poverty on unfavourable TB treatment outcomes. There were reports of some infants being exposed to alcohol, illicit drugs, and tobacco which may have occurred during either the intrauterine period or breastfeeding, since information for children is usually given by their parents and/or carers. Finally, the Sinan may include potential errors in data entry, which can occur randomly and would not jeopardise the analyses, as they are continually reviewed to maintain the robustness of the information. Nonetheless, it is noteworthy that numerous studies are conducted and published utilizing data from Sinan.

In conclusion, our study presents the first nationally representative investigation of unfavourable treatment outcomes in children and adolescents with TB in Brazil over 22 years. Our findings indicate that non-white children and adolescents, those with alcohol, illicit drug, or tobacco use, individuals with HIV infection, and those without DOT treatment supervision by a healthcare worker were at a higher risk of experiencing unfavourable treatment outcomes. Conservely, cash transfers appeared protective against death in children and adolescents with TB. TB in children and adolescents can still be challenging, and successful outcomes depend on factors like early diagnosis, access to appropriate healthcare, and adherence to treatment regimens. Additionally, public health efforts to prevent TB transmission and improve overall living conditions can further reduce the prevalence of the disease in all age groups.

## Contributors

JRCH, JRSS, TW and VSS contributed to conceptualisation and study design. JRCH, JRSS and RDSG-N contributed to data acquisition. JRSS, TW and VSS contributed to data analysis and interpretation. JRCH, JRSS, RDSG-N and VSS drafted the manuscript. ELNM, FD, JNBSJ, WACCC, JRLS, RQG and TW contributed to the critical revision of the manuscript. VSS provided administrative, technical, or material support, as well as supervision and mentorship. Each author contributed important intellectual content during manuscript drafting or revision and accepts accountability for the overall work by ensuring that questions pertaining to the accuracy or integrity of any portion of the work are appropriately investigated and resolved. VSS takes responsibility for the fact that this study has been reported honestly, accurately, and transparently; that no important aspects of the study have been omitted, and that any discrepancies from the study as planned have been explained. JRSS and VSS had full access to all the data in the study, and have verified the accuracy of all underlying data. All authors had final responsibility for the decision to submit the manuscript for publication.

## Data sharing statement

The data used are public and available from SINAN DATASUS or can be obtained by contacting the corresponding author.

## Declaration of interests

TW is funded by grants from the Wellcome Trust, UK (209075/Z/17/Z), the Department of Health and Social Care (DHSC), the Foreign, Commonwealth & Development Office (FCDO), the Medical Research Council (MRC) and Wellcome, UK (Joint Global Health Trials, MR/V004832/1), Medical Research Council (Public Health Intervention Development “PHIND” Award, MR/Y503216/1), and a Dorothy Temple Cross Tuberculosis International Collaboration Grant from the Medical Research Foundation (MRF-131-0006-RG-KHOS-C0942), UK. VSS, JRCH, RDSGN, ELNM, FDCJ, JNBSJ, WACCC, JRLS, JRSS and RQG have no conflict of interest to declare.

## Appendix A Supplementary data

eTables
