# Supplementary material for: Social and health factors associated with unfavourable treatment outcomes in children and adolescents with drug-sensitive tuberculosis in Brazil: a national retrospective cohort study
Source: Lancet Reg Health Am. 2024 Nov 13;40:100938. doi: 10.1016/j.lana.2024.100938 (PMC11613200; doi:10.1016/j.lana.2024.100938)
Supplement: eTables [file mmc1.docx]

**Appendix**

**Social and health factors associated with unfavourable treatment outcomes in children and adolescents with drug-sensitive tuberculosis in Brazil: a national retrospective cohort study**

**eTable 1. Distribution of the number of children and adolescents who reported using alcohol, illicit drugs and tobacco by age.**

| **Age** | **Alcohol***  **N (%)** | **Illicit drugs***  **N (%)** | **Tobacco***  **N (%)** | **Total in each age group**  **N (%)** |
| --- | --- | --- | --- | --- |
| 0 | 403 (29·6) | 141 (11·0) | 267 (22·3) | 6335 (7·2) |
| 1 | 4 (0·3) | 3 (0·2) | 4 (0·3) | 3584 (4·1) |
| 2 | 5 (0·4) | 1 (0·1) | 3 (0·3) | 2904 (3·3) |
| 3 | 6 (0·4) | 0 (0·0) | 1 (0·1) | 2543 (2·9) |
| 4 | 10 (0·7) | 4 (0·3) | 6 (0·5) | 2309 (2·6) |
| 5 | 8 (0·6) | 3 (0·2) | 1 (0·1) | 2228 (2·5) |
| 6 | 11 (0·8) | 2 (0·2) | 2 (0·2) | 2113 (2·4) |
| 7 | 8 (0·6) | 1 (0·1) | 3 (0·3) | 2030 (2·3) |
| 4 | 6 (0·4) | 0 (0·0) | 1 (0·1) | 2060 (2·3) |
| 9 | 7 (0·5) | 1 (0·1) | 0 (0·0) | 2054 (2·3) |
| 10 | 9 (0·7) | 2 (0·2) | 4 (0·3) | 2378 (2·7) |
| 11 | 19 (1·4) | 4 (0·3) | 3 (0·3) | 2557 (2·9) |
| 12 | 16 (1·2) | 7 (0·5) | 2 (0·2) | 3168 (3·6) |
| 13 | 26 (1·9) | 20 (1·6) | 10 (0·8) | 4508 (5·1) |
| 14 | 54 (4·0) | 52 (4·1) | 43 (3·6) | 6759 (7·7) |
| 15 | 121 (8·9) | 151 (11·8) | 132 (11·0) | 9804 (11·1) |
| 16 | 230 (16·9) | 296 (23·2) | 253 (21·2) | 13664 (15·5) |
| 17 | 418 (30·7) | 590 (46·2) | 460 (38·5) | 17272 (19·6) |

*****As parents and/or guardians are usually responsible for providing information about children, we interpreted exposure in younger ages as possibly occurring during the intrauterine period, breastfeeding, or through any inappropriate use (even if a small dose of exposure). Additionally, there may have been data entry errors when filling out the notification form, which could have occurred randomly across the Sinan system.

**eTable 2.** **Proportion of missing data between favourable and unfavourable tuberculosis treatment outcomes.**

| **Variable** | **Favourable treatment**  **Outcome**  **N = 78967**  **N (%)** | **Unfavourable treatment outcome**  **N = 9303**  **N (%)** |
| --- | --- | --- |
| Sex | 16 (0·02) | 3 (0·03) |
| Ethnicity (skin colour) | 15029 (19·0) | 1895 (20·3) |
| Region | 65 (0·1) | 10 (0·1) |
| Government cash transfer | 3951 (5·0) | 653 (7·0) |
| Deprivation of liberty | 1292 (1·6) | 177 (1·9) |
| Homelessness | 1323 (1·7) | 200 (2·1) |
| Alcohol use | 17150 (21·7) | 1994 (21·4) |
| Illicit drug use | 1512 (1·9) | 251 (2·6) |
| Tobacco use | 1418 (1·8) | 248 (2·6) |
| HIV infection | 37949 (48·0) | 5150 (55·0) |
| Diabetes | 17441 (22·0) | 2038 (22·0) |
| Clinical feature of tuberculosis | 0 (0·0) | 1 (0·0) |
| Directly observed therapy | 3973 (5·0) | 633 (6·8) |

**eTable 3. Multivariate Logistic Regression Model (Missing Indicator Analysis) estimating the association between Characteristics of Children and Adolescents with Tuberculosis in Brazil and Unfavourable TB Treatment Outcomes**

| **Variable** | **Missing Indicator Analysis**  **Adjusted Odds Ratio (95% CI)** | **P–value** |
| --- | --- | --- |
| **Age group** |  |  |
| Children | Reference |  |
| Adolescents | 1·0 (1·0-1·1) | 0·104 |
| **Sex** |  |  |
| Male | 1·1 (1·1-1·2) | 0·000 |
| Female | Reference |  |
| **Ethnicity (skin colour)** |  |  |
| Asian | 1·5 (1·1-1·8) | 0·003 |
| Indigenous | 1·3 (1·1-1·5) | 0·000 |
| Brown (*Pardo*) | 1·5 (1·4-1·6) | 0·000 |
| Black | 1·6 (1·5-1·8) | 0·000 |
| White | Reference |  |
| **Region** |  |  |
| Central West | 0·9 (0·8-1·1) | 0·221 |
| Northeast | 1·0 (0·9-1·0) | 0·328 |
| North | 0·9 (0·8-1·0) | 0·103 |
| Southeast | 1·0 (0·9-1·0) | 0·400 |
| South | Reference |  |
| **Government cash transfer** |  |  |
| Yes | 0·9 (0·8-1·0) | 0·043 |
| No | Reference |  |
| **Deprivation of liberty** |  |  |
| Yes | 0·8 (0·6-1·1) | 0·121 |
| No | Reference |  |
| **Homelessness** |  |  |
| Yes | 1·9 (1·2-2·7) | 0·003 |
| No | Reference |  |
| **Alcohol use** |  |  |
| Yes | 1·8 (1·5-2·1) | 0·000 |
| No | Reference |  |
| **Illicit drug use** |  |  |
| Yes | 3·4 (2·9-3·9) | 0·000 |
| No | Reference |  |
| **Tobacco use** |  |  |
| Yes | 1·5 (1·3-1·8) | 0·000 |
| No | Reference |  |
| **HIV infection** |  |  |
| Yes | 3·0 (2·7-3·4) | 0·000 |
| No | Reference |  |
| **Diabetes** |  |  |
| Yes | 1·0 (0·8-1·3) | 0·797 |
| No | Reference |  |
| **Clinical feature of tuberculosis** |  |  |
| Pulmonary | 1·2 (1·1-1·2) | 0·000 |
| Pulmonary + Extrapulmonary | 1·7 (1·5-1·9) | 0·000 |
| Extrapulmonary | Reference |  |
| **Directly observed therapy** |  |  |
| Yes | Reference |  |
| No | 2·2 (2·1-2·3) | 0·000 |

**eTable 4. Multivariate Logistic Regression Models estimating the association between Characteristics of Children and Adolescents in Brazil and Unfavourable Tuberculosis Treatment Outcomes (Missing Indicator Analysis)**

|  | **Children** | | **Adolescents** | |
| --- | --- | --- | --- | --- |
| **Variable** | **Missing Indicator Analysis**  **Adjusted Odds Ratio (95% CI)** | **P–value** | **Missing Indicator Analysis**  **Adjusted Odds Ratio (95% CI)** | **P–value** |
| **Sex** |  |  |  |  |
| Male | 1·1 (1-1·2) | 0·102 | 1·1 (1·1-1·2) | 0·000 |
| Female | Reference |  | Reference |  |
| **Ethnicity (skin colour)** |  |  |  |  |
| Asian | 1·7 (1·1-2·6) | 0·022 | 1·4 (1·1-1·9) | 0·018 |
| Indigenous | 1·3 (1·1-1·6) | 0·009 | 1·3 (1·1-1·7) | 0·008 |
| Brow (*Pardo*) | 1·3 (1·2-1·5) | 0·000 | 1·5 (1·4-1·6) | 0·000 |
| Black | 1·5 (1·3-1·7) | 0·000 | 1·7 (1·6-1·9) | 0·000 |
| White | Reference |  | Reference |  |
| **Region** |  |  |  |  |
| Central West | 0·9 (0·7-1·1) | 0·418 | 1·0 (0·8-1·2) | 0·764 |
| Northeast | 1·3 (1·1-1·6) | 0·001 | 0·8 (0·7-0·9) | 0·000 |
| North | 1·2 (1·0-1·4) | 0·060 | 0·8 (0·7-0·9) | 0·001 |
| Southeast | 1·0 (0·9-1·2) | 0·748 | 0·9 (0·9-1·0) | 0·260 |
| South | Reference |  | Reference |  |
| **Government cash transfer** |  |  |  |  |
| Yes | 1 (0·8-1·2) | 0·724 | 0·8 (0·7-1·0) | 0·022 |
| No | Reference |  | Reference |  |
| **Deprivation of liberty** |  |  |  |  |
| Yes | 0·5 (0·3-0·8) | 0·007 | 1·1 (0·7-1·5) | 0·787 |
| No | Reference |  | Reference |  |
| **Homelessness** |  |  |  |  |
| Yes | 2·1 (1·1-3·9) | 0·025 | 1·9 (1·1-3·1) | 0·015 |
| No | Reference |  | Reference |  |
| **Alcohol use** |  |  |  |  |
| Yes | 1·8 (1·3-2·3) | 0·000 | 1·8 (1·5-2·2) | 0·000 |
| No | Reference |  | Reference |  |
| **Illicit drug use** |  |  |  |  |
| Yes | 1·8 (1·2-2·8) | 0·008 | 3·5 (3·0-4·2) | 0·000 |
| No | Reference |  | Reference |  |
| **Tobacco use** |  |  |  |  |
| Yes | 1·5 (1·1-2·2) | 0·016 | 1·6 (1·3-1·9) | 0·000 |
| No | Reference |  | Reference |  |
| **HIV infection** |  |  |  |  |
| Yes | 2·7 (2·2-3·2) | 0·000 | 3·4 (2·9-3·9) | 0·000 |
| No | Reference |  | Reference |  |
| **Diabetes** |  |  |  |  |
| Yes | 0·9 (0·6-1·2) | 0·433 | 1·1 (0·8-1·5) | 0·401 |
| No | Reference |  | Reference |  |
| **Clinical feature of tuberculosis** |  |  |  |  |
| Pulmonary | 1·0 (0·9-1·1) | 0·723 | 1·3 (1·2-1·4) | 0·000 |
| Pulmonary + Extrapulmonary | 1·7 (1·4-2·0) | 0·000 | 1·7 (1·5-2·1) | 0·000 |
| Extrapulmonary | Reference |  | Reference |  |
| **Directly observed therapy** |  |  |  |  |
| Yes | Reference |  | Reference |  |
| No | 2·2 (2·0-2·4) | 0·000 | 2·2 (2·0-2·4) | 0·000 |

**eTable 5. Multinomial logistic regression model of factors associated with the different components of unfavourable outcomes in tuberculosis treatment among Brazilian children and adolescents with TB treatment success (cure and treatment completion) as the reference outcome (Missing Indicator Analysis).**

| **Variable** | **Loss to follow up** | | **Death** |  |
| --- | --- | --- | --- | --- |
|  | **Missing Indicator**  **Analysis**  **Adjusted RRR**  **(95% CI)** | **P–value** | **Missing Indicator**  **Analysis**  **Adjusted RRR**  **(95% CI)** | **P–value** |
| **Age group** |  |  |  |  |
| Children | 0·9 (0·8-0·9) | 0·000 | 1·7 (2·7-2·8) | 0·000 |
| Adolescents | Reference |  | Reference |  |
| **Sex** |  |  |  |  |
| Male | 1·1 (1·1-1·2) | 0·000 | 0·9 (1·7-1·8) | 0·000 |
| Female | Reference |  | Reference |  |
| **Ethnicity (skin colour)** |  |  |  |  |
| Asia | 1·5 (1·5-1·5) | 0·000 | 1·7 (0·3-0·4) | 0·000 |
| Indigenous | 1·1 (1·1-1·2) | 0·000 | 2·2 (1·7-1·7) | 0·000 |
| Brown (*Pardo*) | 1·4 (1·4-1·5) | 0·000 | 0·9 (2·1-2·2) | 0·000 |
| Black | 1·7 (1·6-1·8) | 0·000 | 1·1 (0·9-0·9) | 0·001 |
| White | Reference |  | Reference |  |
| **Region** |  |  |  |  |
| Central West | 0·9 (0·8-0·9) | 0·000 | 1·3 (1·2-1·3) | 0·000 |
| Northeast | 0·9 (0·9-1·0) | 0·002 | 0·2 (1·3-1·3) | 0·000 |
| North | 0·9 (0·8-0·9) | 0·000 | 2·3 (0·2-0·2) | 0·000 |
| Southeast | 1·0 (0·9-1·0) | 0·140 | 2·3 (2·2-2·4) | 0·000 |
| South | Reference |  | Reference |  |
| **Government cash transfer** |  |  |  |  |
| Yes | 0·9 (0·9-0·9) | 0·000 | 2·4 (2·4-2·4) | 0·000 |
| No | Reference |  | Reference |  |
| **Deprivation of liberty** |  |  |  |  |
| Yes | 0·9 (0·9-0·9) | 0·000 | 1·2 (1·2-1·2) | 0·000 |
| No | Reference |  | Reference |  |
| **Homelessness** |  |  |  |  |
| Yes | 1·7 (1·7-1·7) | 0·000 | 1·0 (1·0-1·0) | 0·000 |
| No | Reference |  | Reference |  |
| **Alcohol use** |  |  |  |  |
| Yes | 1·7 (1·7-1·7) | 0·000 | 1·0 (1·0-1·0) | 0·000 |
| No | Reference |  | Reference |  |
| **Illicit drug use** |  |  |  |  |
| Yes | 3·6 (3·6-3·7) | 0·000 | 0·8 (0·8-0·8) | 0·000 |
| No | Reference |  | Reference |  |
| **Tobacco use** |  |  |  |  |
| Yes | 1·6 (1·6-1·6) | 0·000 | 1·4 (1·4-1·4) | 0·000 |
| No | Reference |  | Reference |  |
| **HIV infection** |  |  |  |  |
| Yes | 2·8 (2·7-2·8) | 0·000 | 1·0 (1·0-1·0) | 0·000 |
| No | Reference |  | Reference |  |
| **Diabetes** |  |  |  |  |
| Yes | 0·8 (0·8-0·8) | 0·000 | 0·9 (0·9-0·9) | 0·000 |
| No | Reference |  | Reference |  |
| **Clinical feature of tuberculosis** |  |  |  |  |
| Pulmonary | 1·2 (1·2-1·3) | 0·000 | 3·2 (3·2-3·2) | 0·000 |
| Pulmonary + Extrapulmonary | 1·3 (1·3-1·3) | 0·000 | 0·7 (0·7-0·7) | 0·000 |
| Extrapulmonary | Reference |  | Reference |  |
| **Directly observed therapy** |  |  |  |  |
| Yes | Reference |  |  |  |
| No | 2·3 (2·2-2·4) | 0·000 | 1·9 (1·9-1·9) | 0·000 |
